# ORAL CONCURRENT SESSION 7: Diabetes

**DOI:** 10.1002/pmf2.12017

**Published:** 2025-01-05

Abstracts 77 – 85

SATURDAY

February 1, 2025

8:00 AM – 10:15 AM

Aurora Ballroom A

MODERATORS

Christina M. Scifres, MD

Lynn M. Yee, MD, MPH

